# Understanding the nexus between public risk perception of COVID-19 and evacuation behavior during cyclone Amphan in Bangladesh

**DOI:** 10.1016/j.heliyon.2021.e07655

**Published:** 2021-07-22

**Authors:** Md. Shaharier Alam, Torit Chakraborty

**Affiliations:** a Asian Disaster Preparedness Center, Bangladesh Country Office, Rajshahi-6202, Bangladesh; b Urban and Rural Planning Discipline, Khulna University, Khulna-9208, Bangladesh

**Keywords:** COVID-19, Risk perception, Cyclone Amphan, Evacuation behavior, Principal component analysis

## Abstract

In May 2020, when Bangladesh was struggling with community transmission of COVID-19, the country had to face the strongest tropical storm- Cyclone Amphan -which puts the evacuation process in jeopardy. Thus, it is crucial to measure the public risk perception about COVID-19 and its influence on the evacuation decision. This study explores the nexus between COVID-19 risk perception and coastal peoples’ evacuation decisions during cyclone Amphan. With an analysis of 378 sample households survey data of the Satkhira district, this study developed the COVID-19 risk perception index using Principal Component Analysis (PCA) and categorized the respondents based on the score. The result shows that 1.85 %, 21.43 %, 45.77 %, 25.13 %, and 5.82 % have very low, low, moderate, high, and very high-risk perceptions, respectively. The analysis also reveals that 96.6 % of the respondents received an evacuation order during Amphan, but only 42 % complied with the order. The t-test analysis and common language effect size test of the survey data reveal that the respondents with a high perception score are 65 % less likely to evacuate than the respondents with low perception scores. This study has important implications in guiding concerned authorities to combat natural disasters during COVID-19 and other similar public health emergencies in the future.

## 1 Introduction

Bangladesh is regarded as the land of disaster as the country experienced more than 200 natural disasters in the last three decades, which causes massive destruction of livelihoods and economy and the deterrent in the path of accomplishing sustainability (Reliefweb, 2020; Alam and Haque, 2018; Alam et al., 2019a, Alam et al., 2019b; Islam et al., 2019; Alam and Haque, 2020). The country's prolonged coastline, which is residence to around 35 million people, gets continuously battered by cyclones and other disasters in the most imperiled way (Karim and Mimura, 2008; Rahman and Rahman, 2015; Islam et al., 2021; Ha-mim et al., 2020; Ha-mim and Hossain, 2021). Cyclone is one of Bangladesh's most recurring disasters due to its unique geophysical setting, triangular-shaped coastline geography, high sea temperature, etc (Paul, 2009; Dasgupta et al., 2014; Islam et al., 2021). Due to utmost effort from the government side, Bangladesh succeeded to reduce the devastation of the cyclone through policy formulation, effective weather forecasting, and early warning system, improved emergency evacuation planning, implementing numerous structural and non-structural measures in the coastal area (Paul and Dutt, 2010; Ahsan et al., 2016a, Ahsan et al., 2016b). Still, many people didn't evacuate in Bangladesh's past cyclones due to public cyclone shelter problems, early warning related issues, socio-economic problems, and non-evacuees’ perceptions (Saha and James, 2017; Paul and Dutt, 2010; Islam et al., 2021; Ahsan et al., 2016a, Ahsan et al., 2016b). On 20 May 2020, Bangladesh had to face the Super Cyclone Amphan, the strongest cyclone ever originated in the Bay of Bengal, with 60–90 Km/h wind speed and high tidal inundation (Ellis-Petersen and Ratcliffe, 2020; Mishra and Vanganuru, 2020; Majumdar and DasGupta, 2020). Though Bangladesh had done a massive evacuation during cyclone Amphan, this cyclone was exceptional from all previous cyclones as an unprecedented dimension of the pandemic was added during that time.

The World Health Organization (WHO) declared the COVID-19 as a pandemic on 12 March 2020 that has dispersed in the whole world within a blink of an eye and creates unprecedented public health and socio-economic challenges (Baker et al., 2020; Ahasan et al., 2020; Ahasan and Hossain, 2020; Rahman et al., 2021). COVID-19 during the time of natural disaster creates double jeopardy, and the world is facing complications in handling disasters while making efforts to decelerate the dispersion of COVID-19 (Shultz et al., 2020; Ishiwatari et al., 2020). Therefore, it is essential to comprehend the impact of COVID-19 on disaster management at the national level and decisional aspects at the individual level. Large-scale evacuation in the time of disaster challenges the physical distancing requirements of lockdowns, making the evacuees susceptible to COVID-19 infection (Collins et al., 2021; Ebrahim et al., 2020; Shammi et al., 2020). On 20 May 2020, when the super cyclone hit Bangladesh, the country was already susceptible with about 21,145 active COVID cases, and the cyclone Amphan created a dual crisis for the country. Thus, the risk perception of coastal people about the COVID-19 pandemic can influence evacuation decisions during the cyclone as it conflicts with the social distancing principles and creates potential infection threats. Therefore, it is of utmost importance to analyze how COVID-19 risk perception of coastal peoples shapes their evacuation decision during cyclone Amphan to provide a perspicuous idea of disaster management during the pandemic.

Since the diffusion of COVID-19, a significant number of researchers tried to connect the COVID-19 infection pattern with different social and behavioral science issues such as inequity (Fortuna et al., 2020; Gray et al., 2020), social vulnerability (Karaye and Horney, 2020; Kim and Bostwick, 2020), housing pattern (Jowers et al., 2021; Benfer et al., 2021), decision analysis considering previous infectious diseases (Southwell et al., 2020; Alamoodi et al., 2020), etc. Risk perception analysis about COVID-19 and its relation to behavioral, socio-economic factors and the public health sector is one of the most explored research areas (Niepel et al., 2020; Samadipour et al., 2020; Bruine de Bruin, 2020; He et al., 2020; Abir et al., 2020; Dryhurst et al., 2020). Understanding how persons perceive and conceptualize a phenomenon is the first step for any public health intervention (Southwell et al., 2018). After the declaration of COVID-19 as a pandemic, many research works were conducted to understand public perception and behavioral response about the pandemic at the local (He et al., 2020; Zhong et al., 2020; Kuang et al., 2020), national (Bruine de Bruin, 2020; Samadipour et al., 2020; Abir et al., 2020) or global scale (Dryhurst et al., 2020). Based on a comprehensive literature review, it is found that some studies mainly focused on assessing risk perception (He et al., 2020; Dryhurst et al., 2020; Samadipour et al., 2020), whereas other studies are more focused on establishing the relationship of COVID-19 risk with other aspects including fatality (Niepel et al.,.2020), protective behavior (de Bruin and Bennett, 2020) or social distancing (Xie et al., 2020), emotional states and mental healthcare (Roy et al., 2015; de Bruin and Bennett, 2020). But none of these existing studies of COVID-19 risk perception tried to understand how people of different risk perceptions behave in a real-time emergency like natural hazards. Though very few studies focused on natural disasters during COVID -19, those are either focused on imminent risk analysis based on previous knowledge of different disasters worldwide. Many studies, including Markotić and Capac (2020); Sawano et al. (2021); Kanamori et al. (2021); Shultz et al. (2020), and Ishiwatari et al. (2020), emphasized considering COVID-19 in disaster studies through discussion and analysis based on previous hazards scenarios. Some studies, such as Pei et al. (2020) and Currie et al. (2020), endeavored to predict the evacuation scenario during natural hazards in the COVID-19 pandemic by developing hypothetical models based on previous disaster data. The only study of Collins et al. (2021) attempted to understand how COVID-19 risk perception influenced the willingness to evacuate coastal people of Florida in any upcoming hurricane. Collins et al. (2021) reported that COVID-19 drastically influenced the evacuation willingness of coastal people, which is very alarming and justifies the need for this research. But no empirical studies are conducted to understand how the COVID-19 pandemic impacted real-time disaster evacuation events. Thus, it is highly important to study whether COVID-19 risk perception can influence people's decisions during an emergency evacuation scenario. To understand this scenario, this study explores the relationship between COVID-19 risk perception and the evacuation decisions of Bangladesh's coastal peoples during a super cyclone Amphan, which occurred during the pandemic. This study's empirical findings are expected to be useful for concerned authorities and policymakers to formulate effective emergency preparedness and mitigation measures for future similar emergency events.

## 2 Concept and context

### 2.1 COVID-19 risk perception

The COVID-19 pandemic, a global public health crisis, involves large-scale behavior change, which puts significant psychological pressure on individuals (Van Bavel et al., 2020). Risk perceptions, an individual's instinctive judgments of susceptibility to unexpected events, have always been crucial in analyzing the dispersion of an endemic (Dryhurst et al., 2020; Cori et al., 2020). People's risk perception on undertaking protective behavior and other decisional aspects can guide public health policymakers in limiting this outbreak (Samadipour et al., 2020; Samadipour et al., 2020). Generally, the research on public risk perception is done in the later period of an endemic when it became a matter of mass concern rather than in the beginning phase (Shabu et al., 2020). Most of the studies used statistical analysis, including descriptive statistics, index development, logistic regression, principal component analysis, confirmatory factor analysis, t-test, etc., to measure the risk perception and its relation with other factors. In most of the cases, the Likert scale was used to collect the data from the respondent. In this study, the risk perception of Bangladesh's coastal peoples is measured first by developing a risk perception index to categorize coastal people based on how they perceive the pandemic. Then, the association of risk perception with the evacuation decision during cyclone Amphan is measured using statistical analysis in this study.

### 2.2 Evacuation scenario in cyclone in Bangladesh

Tropical cyclones hit the low-lying coastal zone of Bangladesh with devastating winds and storm surges almost every year, which cost the loss of life and properties (Islam et al., 2021). Two Cyclone Gorky (1991) and Cyclone Sidr (2007), with category 4 intensity cyclone, caused the death of around 140,000 and 3,400 coastal inhabitants, respectively (Paul, 2009; Saha and James, 2017). Bangladesh efficiently managed to reduce life loss and property damage by cyclones by employing the utmost effort to significantly improve cyclone early warning arrangements and evacuation techniques (Paul, 2009). Still, many people didn't comply with the evacuation order in the past cyclones of Bangladesh due to public cyclone shelter problems, early warning issues, socio-economic issues, and perceptions of the non-evacuee (Saha and James, 2017; Paul and Dutt, 2010). Along with all these factors, as mentioned above, the risk of COVID-19 infection, pandemic, social distancing, and subsequent lookdown added an unprecedented dimension during the cyclone Amphan.

Moreover, cyclone Amphan, a category 5 intensified cyclone, was the fiercest tropical storm, super-cyclone Amphan, ever strike the Ganges Delta of this century (Mishra and Vanganuru, 2020). Considering the intensity of cyclone Amphan and the pandemic's unprecedented challenge, it can be assumed that the evacuation scenario before Amphan is unparalleled with the previous cyclones. Therefore, this study aims to analyze the coastal people's evacuation decision using primary survey data and identify how the risk perception of COVID-19 influenced the evacuation decision by applying statistical analysis.

## 3 Methodology

### 3.1 Study area

**Figure 1:**
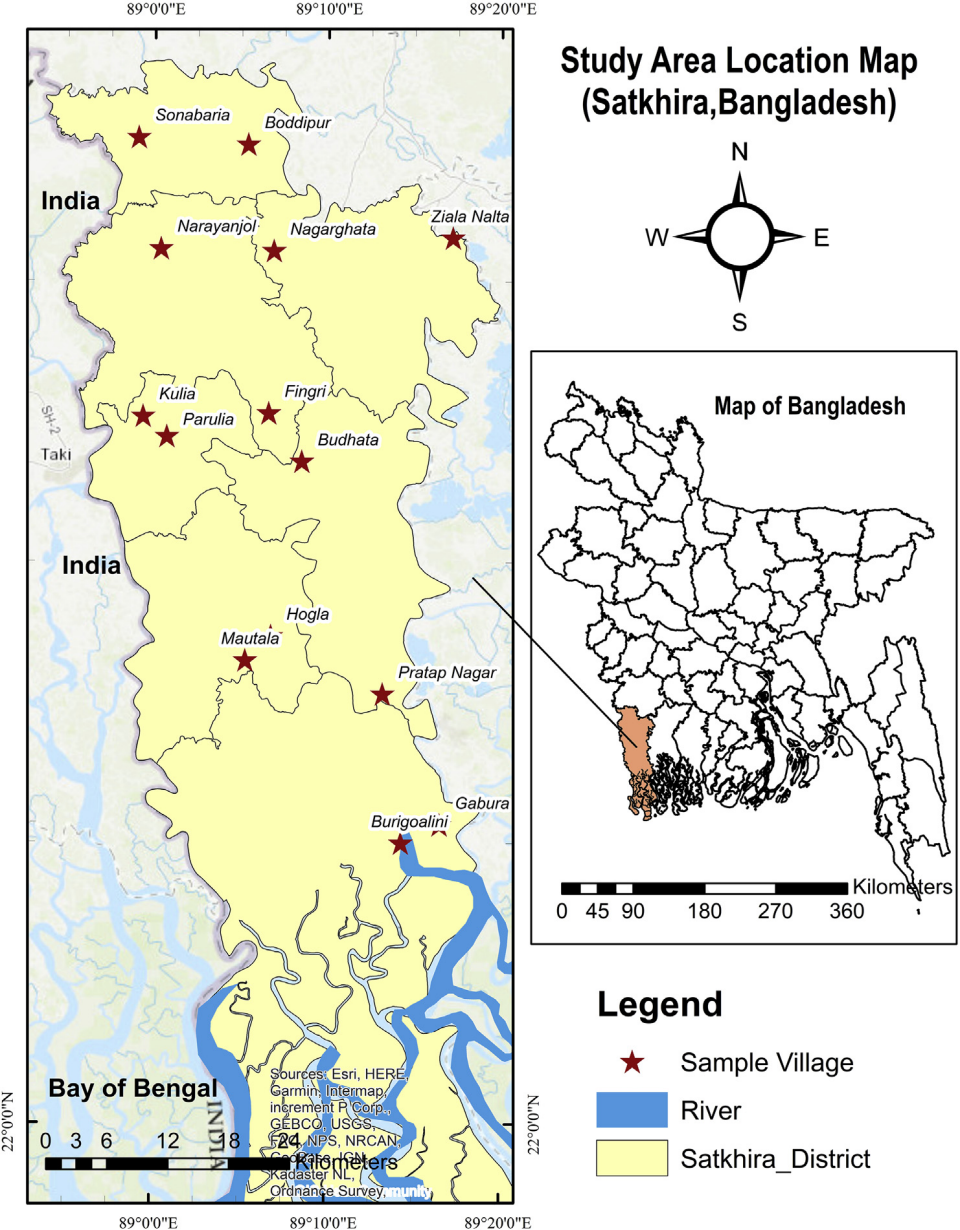
Map of the surveyed villages of Satkhira district (Source: Author, 2020).

Though cyclone Amphan made landfall in West Bengal, India, it barreled into Bangladesh through the Satkhira district with high-speed winds, torrential rain, and high tidal surge (Ellis-Petersen and Ratcliffe, 2020; Mishra and Vanganuru, 2020; Majumdar and DasGupta, 2020). When the strongest tropical cyclone Amphan (Category 5 Hurricane), was intimidating in the Bay of Bengal to wreak deadly havoc, Satkhira district was already dealing with 1617 active COVID-19 cases. So when the evacuation order came during the cyclone Amphan, the district people had to take the daunting decision of compliance or non-compliance with the evacuation order considering the risk of COVID-19 infection. Considering the aforementioned scenario, Satkhira District is selected as the study area for this study. The total administrative area of Satkhira district is 3817.29 sq. km (includes 1632 sq. km. of Sundarbans.) with 7 Upazila, 2 Paurashava, 79 unions, and 1436 villages. As per the last population census of 2011, Satkhira district is home to 754097 people with a population density of 198 per sq. km. The male-female ratio of the Satkhira district is 1.01, and the literacy rate is 52.07 % (BBS, 2011; Alam et al., 2019a, Alam et al., 2019b). We have opted to randomly choose two villages in each Upazila (a total of 14 villages in 7 Upazila) to conduct this research. The selected villages are Boddipur and Sonabaria in Kalaroa Upazila, Nagarghata and Ziala Nalta in Tala Upazila, Narayonjol and Fingri in Satkhira Sadar Upazila, Kulia and Parulia in Debhata Upazila, Budhata and Pratapnagar in Assasuni Upazila, Hogla and Mautala in Kaliganj Upazila, Gabura and Burigoalini in Shaymnagar Upazila of Satkhira district. The location map of the study area is shown in Figure 1.

### 3.2 Indicator selection and questionnaire preparation

**Figure 2:**
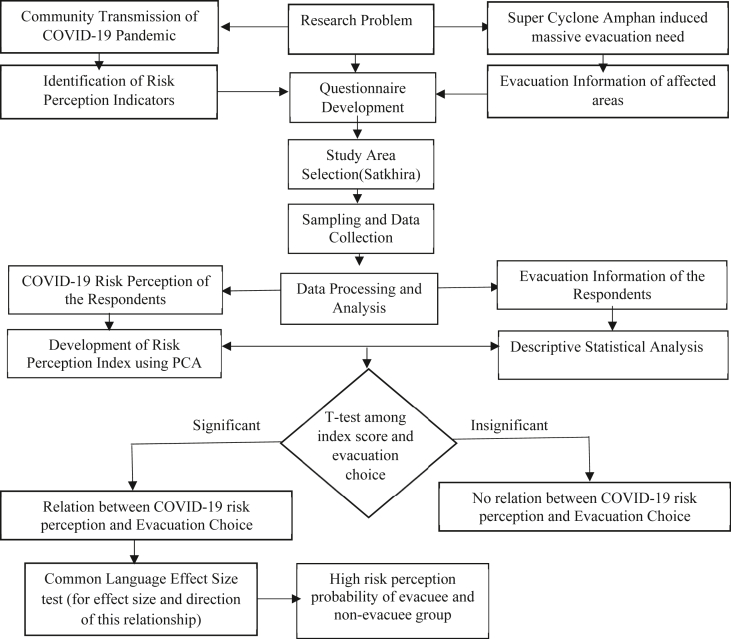
Methodological framework of the study.

A comprehensive questionnaire was designed in this study to measure the relationship between risk perception about COVID-19 and evacuation decisions of coastal peoples during cyclone Amphan. The questionnaire was divided into three parts: the demographic section, COVID-19 risk perception section, and evacuation decision section (See Figure 2). The demographic section includes participants’ demographic and socio-economic information such as age, gender, education, employment, religion, marital status, household type, family type, etc. The next section contains 34 questionnaire items to measure the risk perception, derived from a comprehensive literature review and the World Health Organization (WHO) guideline for safety measures of COVID-19 (Shown in Table 1). The participants assessed the questionnaire items on a 5-point Likert scale ranging from 1 (“completely disagree”) to 5 (“completely agree”). In the last section, the questionnaire item includes the evacuation decision of the participant during cyclone Amphan. As this research is based on the human subject, ethical approval of the questionnaire and study were taken from the Urban Resilience Department of Asian Disaster Preparedness Center, Bangladesh, to ensure anonymity and confidentiality.

**Table 1:** Principal components (PC), variables, loadings, communality, and variance for the COVID-19 risk perception index.

Component Name	Questionnaire Item	Communality	Loading	Variance
Cognitive Factors (PC1)	Are you aware of the Coronavirus disease (COVID-19) outbreak?	0.950	0.946	30.436 %
	Do you think the Coronavirus disease (COVID-19) outbreak is dangerous?	0.862	0.818	
	How much do you recall the symptom of COVID-19?	0.811	0.753	
	Do you think Hand Hygiene/Hand cleaning is important to control the spread of the Coronavirus disease (COVID-19) outbreak?	0.814	0.859	
	Do you think wearing masks is important to control the spread of the Coronavirus disease (COVID-19) outbreak?	0.800	0.828	
	Those that have contact with someone who has COVID-19 infection should be isolated in the right place immediately. The observation period is usually 14 days	0.784	0.761	
	Children and adults should take steps to prevent the COVID-19 virus from infection.	0.671	0.76	
	COVID-19 individuals with no symptoms of fever cannot spread the virus to anyone	0.641	0.683	
	Individuals should stop being crowded to prevent COVID-19 infection.	0.676	0.634	
	How much do you feel you understand the government's strategy to deal with the coronavirus/COVID-19 pandemic?	0.765	0.662	
	Do you have the process of getting tested for COVID-19 (i.e., contract numbers of official, testing location, etc.)?	0.749	0.455	
	It can be treated at home	0.619	0.618	
	I feared that my society would boycott me if I got COVID	0.680	0.501	
	I will not get proper treatment if I get COVID-19	0.677	0.668	
	I am planning to/have already limited my travel plans/doing work from home	0.560	0.517	
Political Factors (PC2)	The politician/policymakers have appropriate knowledge of the COVID-19 pandemic	0.745	0.715	8.875 %
	Do you think Public Health Authorities in Bangladesh are doing enough to control the Coronavirus disease (COVID-19) outbreak?	0.824	0.796	
	How much do you trust the country's politicians to deal effectively with the pandemic?	0.847	0.827	
Protective Behavior (PC3)	To what extent do you feel your country's actions limit the spread of coronavirus makes a difference?	0.650	0.232	7.952 %
	People from the minor religious/cultural group may face discrimination during this pandemic	0.581	0.317	
	To what extent do you feel that the personal actions you are taking to limit coronavirus spread make a difference?	0.920	0.756	
	I plan to/have already taken COVID protection measures (disinfectant, mask, sanitized, hand gloves, washing my hands more, and disinfecting my home.)	0.864	0.764	
	I am planning to/have reduced to shake the hand, avoid crowded space, etc.	0.822	0.638	
Trust Factor (PC4)	Authorities have been negligent in issuing early warnings for COVID-19 disease.	0.926	0.846	6.071 %
	The number of confirmed cases and death is under-reported by the authority	0.918	0.826	
	I am worried/anxious/alarmed and frightened by the quarantine	0.635	0.269	
Fatality Perception Factor (PC5)	Assuming that you have been infected with coronavirus, what do you believe is your likelihood of dying from it?	0.420	0.501	5.997 %
Religious Factor (PC6)	Without doing anything, only relying on God and Only Religious rituals can prevent COVID-19 spread.	0.839	0.313	4.870 %
Willingness Factor (PC7)	Are you willing to carry out prevention measures currently recommended by the authority?	0.670	0.698	4.122 %
Prejudicial Factors (PC8)	Have you heard any rumors regarding the COVID-19 during this pandemic?	0.663	0.668	3.466 %
	COVID-19 is a punishment of God	0.821	0.317	
	Healthcare managers and staff exaggerate the risk of COVID-19	0.847	0.177	
Emotional Factors (PC9)	COVID-19 will NOT affect very many people in the country I'm currently living in	0.666	0.518	2.954 %
	I will probably get sick with the coronavirus/COVID-19	0.696	0.395	

Extraction Method: Principal Component Analysis.

### 3.3 Sampling and data collection

The focus of this study is to find out whether COVID-19 risk perception had an influence on evacuation decisions or not of coastal people during cyclone Amphan in the Satkhira district of Bangladesh. In collecting the primary survey information, this study adopted a systematic sampling method. The sampling procedures are performed in two stages, such as (i) selection of the number of villages; and (ii) determining the appropriate number of interviewees. We finalized the questionnaire through a sequential process such as literature review, discussion with community people, and piloting to check the consistency and uniformity. As the primary survey of this huge population is difficult, a sample size of 378 people was selected using Solvin's formula with a 95 % confidence level and 5 % error margin. To conduct a comprehensive analysis of the risk perception and evacuation decision, randomly fourteen villages (two villages per Upazila) were selected in this study, and the sample size was equally distributed in each village. Therefore, 27 people were randomly surveyed in each village during June 2020, using smartphone-based Kobotoolbox. The content and purpose of this survey were explained to the respondents, and consent was taken before starting the questionnaire survey. As countrywide lockdown and restriction on transport movement was the major challenge of collecting data, and therefore, a ‘random walk’ practice, suggested by World Health Organization (WHO, 2011), was applied for data collection from the field.

### 3.4 Methods

#### 3.4.1 Developing COVID-19 risk perception index using principal component analysis

The methodological approach of this research can be divided into two stages. In the first stage, to measure the participants’ COVID-19 risk perception, an index was developed using Principal Component Analysis (PCA). The assessment of public risk perception depends on multiple sets of different parameters, and creating an index combining all the parameters is quite difficult and time-consuming. Most of the existing literature on COVID-19 risk perception used many parameters such as Samadipour et al. (2020) used 30 parameters, Abir et al. (2020) used 23 parameters, Dryhurst et al. (2020) used 18 parameters. Principal Component Analysis is widely used for handling this huge dataset as a dimension reduction tool that determines each potentiality of parameters and their confidence level in large datasets (Islam et al., 2020).

In the second stage, a hypothesis test was conducted using the t-test to measure the COVID risk perception score's association with the evacuation decision (See Figure 2). In the first stage, 34 variables related to COVID-19 risk were chosen to develop the COVID-19 risk perception index. The data of all variables were collected on a 5 point Likert scale. Principal component analysis (PCA) was used in the dataset to generate a set of independent factors in SPSS software. PCA is a well-known statistical method used for dimensionality-reduction of a large dataset to underscore variation and bring out strong patterns in a dataset by identifying a smaller number of components (Abdi and Williams 2010). PCA increases the interpretability of a large dataset without minimizing information loss by creating new uncorrelated variables (Jolliffe and Cadima, 2016). In this study, Kaiser Normalization and Varimax rotation were applied as extraction methods for extracting the eigenvalue of the components using SPSS software. The components with eigenvalues of 1 or greater than 1 were extracted and assigned cardinality (±). The COVID-19 Risk Perception index was calculated by adding all the component's scores and weighting each component equally to gain a total score (Aksha et al., 2019; Rabby et al., 2020). In this research, the COVID-19 risk perception index for coastal people of Satkhira district was calculated for each participant by adding the principal component values:

(1) COVID-19 Risk Perception Index = PC1+ PC2+PC3+PC3+PC5+PC6+ PC7+PC8+PC9

Here, PC1…. PC9 represents the Principal Component's value.

#### 3.4.2 Statistical analysis between risk perception index score and evacuation choice

To understand the connection between an individual's risk perception about COVID-19 and evacuation decision during cyclone Amphan, two types of statistical tests, including independent sample t-test and common language effect size test, were applied in this study. In the first step, the independent sample t-test was used to determine whether the COVID-19 risk perception affects the target population's evacuation behavior or two groups are different from one another. The t-test is a parametric test sample means of a continuous variable of two independent groups. In this study, COVID-19 risk perception of the sample population was converted into a continuous index value using principal component analysis. From the questionnaire survey, the respondents were classified into two categories, including evacuee and non-evacuee. Finally, using SPSS software, the independent sample t-test was applied to compare the mean of risk perception value of evacuee and non-evacuee groups. Based on the significance level (*p*-value) of the computed t-test result, the connection between the COVID-19 risk perception and evacuation group was determined (See Figure 2).

But the t-test analysis can only identify whether there is any relationship between COVID-19 risk perception and evacuation decision or not. But it cannot describe the type of relationship and its effect size on the sample population as a larger p-value doesn't necessarily mean a strong relationship between two groups (Rosnow and Rosenthal, 2009; Rosenthal et al., 2000). Thus, many research studies, including Parker and Hagan-Burke (2007); Rosnow and Rosenthal (2009); Rosenthal et al. (2000); and Rutledge and Loh (2004), suggested the application of effect size tests in behavioral and social science research to precisely understand the size and direction of the relationship. Considering the importance, this study used an effect size test to understand the effect of COVID-19 risk perception on the two population groups (i.e., evacuee and non-evacuee). To measure this relationship's effect size and direction, the Common Language Effect Size test is applied in this study as it is easier to comprehend than other traditional effect size tests (Brooks et al., 2014; Lakens, 2013). This method was proposed by McGraw and Wong (1992) to measure the probability of superiority of one randomly chosen sample from one group than a randomly sampled score from the other group (Somma et al., 2020; Lakens, 2013). Mathematically, the common language effect size is the probability of obtaining Z score greater than the computed value that corresponds to a difference between groups of 0 in a normal distribution curve (McGraw and Wong, 1992; Lakens, 2013). Z can be calculated by:

(2) $$Z=\frac{\left|\text{M}1-\text{M}2\right|}{\sqrt{\frac{SD_{1}^{2}-SD_{2}^{2}}{2}}}$$

## 4 Analysis and result

Principal Component Analysis was employed in this study to develop the COVID-19 risk perception index by analyzing Likert scale data of 34 variables. The initial scrutiny of the *R*-matrix pointed to a substantial number of the coefficients were above .30. The Kaiser-Mayer-Olkin (KMO) index was 0.836, greater than the recommended value of 0.6 (Kaiser, 1970). Bartlett's Test of Sphericity (Bartlett, 1954) also achieved statistical significance (*χ*2 = 10634.565, *p* < .001), indicating that the collected data were appropriate for factor analysis. The communalities were also checked, and factors with communalities of more than 0.3 were included (Shown in Table 1). The preliminary analysis results uncovered nine components with eigenvalues greater than 1, explaining 74.74 % of the total variance. The first principal component (PC1) interpreted 30.44 % of the total variance, encompassing a significant level of cognitive factors such as knowledge of COVID-19 awareness, symptoms, hand cleaning, mask-wearing, government strategy quarantines, etc. The second principal component (PC2) explained 8.88 % of the variance, including political factors such as public perception of politicians and public health officials' knowledge to deal with the pandemic effectively. The third component (PC3) represented 7.95 % of total variances, which significantly focused on the protective behavior measures of the respondents. The fourth component (PC4) explained 6.05 % of the variance and mainly included public trust factors on the authorities. Similarly, the next subsequent components including PC5, PC6, PC7, PC8 and PC9 elucidated 5.99 %, 4.87 %, 4.12 %, 3.47 %, and 2.95 % of the variance, respectively (shown in Table 1).

### 4.1 Association of socio-economic factors with COVID-19 risk perception

**Figure 3:**
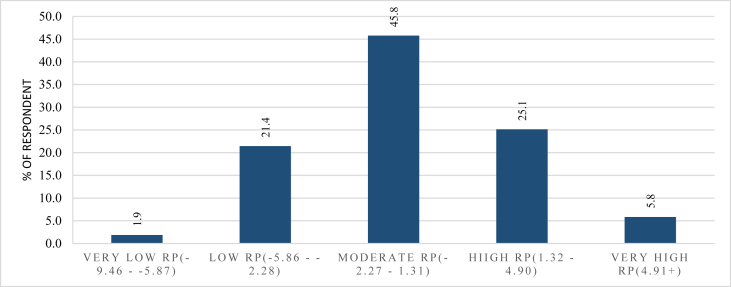
Risk perception categories of coastal people about COVID-19.

**Figure 4:**
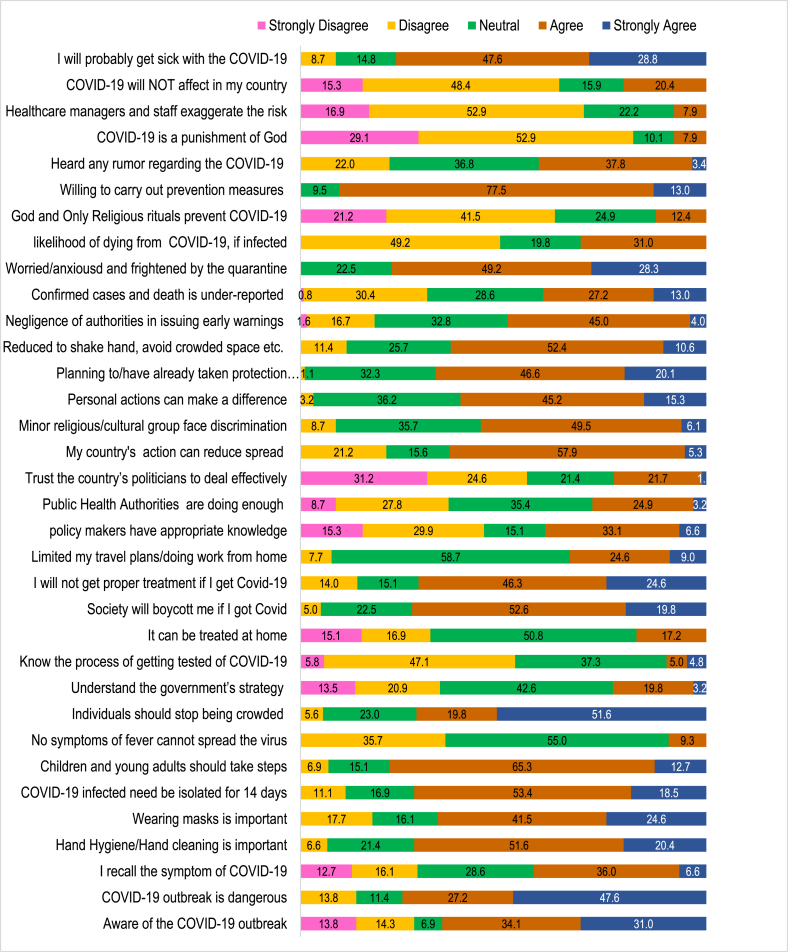
Risk perception of coastal people about the factors of COVID-19 based on Likert Scale data.

Risk perception is a subjective judgment of an individual shaped by rational, emotional, social, cultural, and personal differences (Douglas and Wildavsky 1983; Dryhurst et al., 2020). As the confirmed cases and fatalities are increasing exponentially worldwide, it is essential to recognize the people's risk perception regarding the COVID-19 and its influence on every aspect of life (Van Bavel et al., 2020). In this study, the perception of coastal inhabitants regarding COVID-19 risk was collected on a 5 point Likert scale (strongly disagree, disagree, neutral, agree, and strongly agree) to calculate the risk perception. Figure 4 visualizes what people perceive about different factors of COVID-19, which will be helpful to delineate the gaps and scope of improvements for public health officials. Figure 4 shows that many people in coastal Bangladesh still don't know much about COVID-19 and have many misbeliefs about the pandemic. Then, the COVID-19 risk perception score of the people in coastal areas of Bangladesh was determined using PCA analysis to understand its influence on evacuation decisions during cyclone Amphan. Later, the score was classified into five categories based on the equal interval: very low-risk perception, low-risk perception, moderate risk perception, high-risk perception, and very high-risk perception. The high-risk perception value indicates that the individual is more concerned about COVID-19 than the others. According to analysis, it is revealed that 1.85 % of participants have a very low-risk perception, 21.43 % have a low-risk perception, 45.77 % have moderate risk perception, 25.13 % have a high-risk perception, and 5.82 % of participants has a very high-risk perception about COVID-19 (Figure 3).

Descriptive statistics analysis was also conducted on the collected data to understand the participants' characteristics. The descriptive analysis (Table 2) of the data indicates that the majority of the respondents are male (70.1 %), Muslim (94.7 %), and married (97.4 %). The respondents' mean household size is 4.67, slightly higher than the latest census database (4.21) (BBS, 2011). About 33.3 % of participants have no basic education, and the respondents' average income is 12669 taka^1^. The chi-square test for independence with α = .05 was employed to measure how COVID-19 risk perception varies along with demographic factors. According to the chi-square test result, the participant's age, education, and income have a statistically significant relationship with the COVID-19 risk perception. To measure how strongly these variables are related to the COVID-19 risk perception, the Phi coefficient and Cramer's V analysis were applied to this study using the COVID-19 risk perception index score. Phi coefficient is a measure for the strength of the relationship between two categorical variables in a 2 × 2 contingency table (Prematunga, 2012).

**Table 2:** Socio-economic characteristics of the respondents and their association with COVID-19 risk perception.

Socio-Economic Variable	Category	Percentage	N	Chi-square	Effect Size (Cramer's V/Phi)
Age	18yr than 30 yr	15.1 %	57	χ2 = 22.994, df = 3, sig = 0.001∗∗	0.247
	30 yr to 45 yr	51.3 %	194		
	45yr to 60 yr	27.8 %	105		
	Greater than 60 year	5.8 %	22		
Gender	Male	70.1 %	265	χ2 = 0.013, df = 1, sig = 0.11	0.006
	Female	29.9 %	113		
Education	Illiterate	33.3 %	126	χ2 = 96.252, df = 6, sig = 0.001∗∗	0.505
	Class I–V	24.1 %	91		
	Class VI-X	19.8 %	75		
	SSC or Equivalent	10.8S%	41		
	HSC or Equivalent	8.7 %	33		
	Honors or Equivalent	2.6 %	10		
	Masters or Equivalent	0.5 %	2		
Religion	Muslim	94.7 %	358	χ2 = 0.934, df = 2, sig = 0.627	0.05
	Hindu	4.8 %	18		
	Christian	0.5 %	2		
Income	<5000 BDT	0.8 %	3	χ2 = 18.516, df = 3, sig = 0.001∗∗	0.221
	5000-10000 BDT	33.3 %	126		
	10000 to 20000 BDT	64.0 %	242		
	20000 to 30000 BDT	1.9 %	7		

Note: N=378, Significant variables are marked with (∗∗).

In contrast, Cramer's V is an alternative to Phi coefficient in tables bigger than 2 × 2 tabulation (Grimm, 1993). Akoglu (2018) classified the Phi coefficient and Cramer's V value, where values more than 0.25, 0.15 to 0.25, 0.1 to 0.15, and 0.5 to 0.1 indicate a very strong, strong, moderate, and weak association, respectively. According to the Phi/Cramer's V value shown in Table 2, the respondent's education is strongly associated with the COVID-19 risk perception. In contrast, age and income have strong and moderate associations, respectively.

### 4.2 Evacuation decision of the respondents during cyclone Amphan

Bangladesh, widely known as “ground zero for climate change and disaster’, is prone to tropical cyclones as around 10 % of the world's tropical storms originate in the Indian Ocean and the Bay of Bengal every year (Ahsan et al., 2016a, Ahsan et al., 2016b). The country's funnel-shaped coastline and low elevation are responsible for the high elevated storm surge, which causes 85 % of the cyclone damage worldwide (Haque, 1997). With the Bangladesh government's utmost effort in improving preparedness measures and early warning, the country successfully reduced the number of deaths and damage by tropical cyclones (Ahsan et al., 2020). But the evacuation rates in the previous disasters of Bangladesh are not satisfactory, and a huge percentage of people were always non-compliant with the Bangladesh Meteorological Department's evacuation order (Ahsan et al., 2016a, Ahsan et al., 2016b). Therefore, to understand the evacuation scenario during cyclone Amphan, this study's questionnaire comprises two major research questions: ‘how many people got the evacuation order during Amphan’ and ‘how many people showed compliance with the evacuation order and evacuated to a safer place.’

In Bangladesh, evacuation order during a cyclone is provided by Bangladesh Meteorological Department (BMD) at the last stage, which is at least 10 hours before the predicted landfall (Parvin et al., 2019; Paul, 2009; Ahsan et al., 2020). In the previous cyclones of Bangladesh, 26 % of people got evacuation orders during cyclone Gorky (Chowdhury et al., 1993), 75 % of coastal people got evacuation orders during cyclone Sidr (Paul, 2009), and 97 % of people got orders during cyclone Aila (Ahsan et al., 2016a, Ahsan et al., 2016b). In this study, the respondents were also asked whether they received any evacuation orders during cyclone Amphan or not. According to the field investigation and analysis, it is identified that 96.6 % of the respondent in the Satkhira district got the evacuation order during cyclone Amphan, which depicted the improvement of the cyclone early warning system of Bangladesh. It is also found from the field data analysis that 42.06 % of respondents showed compliance with the evacuation order and evacuated to a safe place during cyclone Amphan.

The evacuation behavior of a person depends on wide-ranging factors. Previous studies on cyclone evacuation in coastal Bangladesh, including Ahsan et al., 2016a, Ahsan et al., 2016b; Parvin et al. (2019); Saha and James (2017); Paul and Dutt (2010); Uddin (2010), Paul (2009), reported several socio-economic factors that influenced the evacuation decision. This study opted to analyze whether the factors of the previous cyclones also influenced evacuation decisions during Cyclone Amphan or not. Thus, the Chi-square test and Cramer's V/Phi test were applied to understand the relationship of these factors with evacuation decisions in cyclone Amphan. The data's statistical analysis identified that age, education, occupation, household type, cattle ownership, and elderly family member factors of the respondents have a significant relationship with the evacuation decision (Shown in Table 3). The descriptive statistics and Cramers V test show that the respondents' household type had a very strong relationship with evacuation decisions, and the people who live in Katcha and wooden houses are more likely to evacuate during cyclone Amphan. Education also has a very strong connection with evacuation decisions, and people with a higher educational background are less likely to evacuate during a cyclone. Education is also found to have significant relation with COVID-19 risk perception. The respondents' age, occupation, and cattle ownership also have a strong relationship with evacuation decisions during cyclone Amphan. But the gender, religion, income, marital status, and family size are found insignificant in the evacuation decision during cyclone Amphan.

**Table 3:** The contrast between respondent evacuation status and socio-demographic profile.

Indicator		Evacuee	Non-evacuee	Chi-square	Effect Size (Cramer's V/Phi)
Age	Less than 30 yr	21 (36.8 %)	36 (63.2 %)	χ2 = 16.991, df = 3, sig = 0.001∗∗	V = 0.212
	30 yr to 45 yr	66 (34 %)	128 (66 %)		
	45yr to 60 yr	60 (57.1 %)	45 (42.9 %)		
	Greater than 60 yr	12 (54.5 %)	10 (45.5 %)		
Gender	Male	107 (40.4 %)	158 (59.6 %)	χ^2^ = 1.034, df = 1, sig = 0.309	ϕ = -.052
	Female	52 (46.0 %)	61 (54.0 %)		
Religion	Muslim	148 (41.3 %)	210 (58.7 %)	χ^2^ = 4.209, df = 2, sig = 0.122	V = 0.106
	Hindu	11 (61.1 %)	7 (38.9 %)		
	Christian	0 (0 %)	2 (100 %)		
Education	Illiterate	67 (53.2 %)	59 (46.8 %)	χ2 = 24.683, df = 6, sig = 0.001∗∗	V = 0.256
	Class I–V	41 (45.1 %)	50 (54.9 %)		
	Class VI-X	30 (40 %)	45 (60 %)		
	SSC or Equivalent	10 (24.4 %)	31 (75.6 %)		
	HSC or Equivalent	5 (15.2 %)	28 (84.8 %)		
	Honors or Equivalent	6 (60 %)	4 (40 %)		
	Masters or Equivalent	0 (0 %)	2 (100 %)		
Number of Family Member	3 Member	16 (50 %)	16 (50 %)	χ2 = 1.087, df = 3, sig = 0.780	V = 0.054
	4 Member	61 (42.7 %)	82 (57.3 %)		
	5 Member	54 (40.6 %)	79 (59.4 %)		
	>5 Member	28 (40 %)	42 (60 %)		
Marital Status	Married	153 (41.6 %)	215 (58.4 %)	χ2 = 5.054, df = 2, sig = 0.08	V = 0.116
	Unmarried	0 (0 %)	2 (100 %)		
	Widow/Divorced	6 (75 %)	2 (25 %)		
Occupation	Agriculture/Farming	49 (53.8 %)	42 (46.2 %)	χ2 = 13.960, df = 3, sig = 0.003 ∗∗	V = 0.192
	Business	61 (45.9 %)	72 (54.1 %)		
	Service	34 (35.4 %)	62 (64.6 %)		
	Others	15 (25.9 %)	43 (74.1 %)		
Household Type	Pucca	3 (6.3 %)	45 (93.8 %)	χ2 = 100.651, df = 3, sig = 0.001∗∗	V = 0.516
	Semi-Pucca	30 (21.4 %)	110 (78.6 %)		
	Katcha	102 (63 %)	60 (37 %)		
	Wooden House	24 (85.7 %)	4 (14.3 %)		
Cattle Ownership	Yes	124 (48.1 %)	134 (51.9 %)	χ2 = 11.999, df = 1, sig = 0.001∗∗	ϕ = 0.178
	No	35 (29.2 %)	85 (70.8 %)		
Child Below 6 yr	Yes	51 (40.5 %)	75 (59.5 %)	χ2 = 0.195, df = 1, sig = 0.658	ϕ = -0.23
	No	108 (42.9 %)	144 (57.1 %)		
Old (60+)	Yes	65 (31.7 %)	140 (68.3 %)	χ2 = 19.712, df = 1, sig = 0.001∗∗	ϕ = -0.228
	No	94 (54.3 %)	79 (45.7 %)		
Income	<5000 BDT	0 (0 %)	3 (100 %)	χ2 = 7.467, df = 3, sig = 0.058	V = 0.141
	5000-10000 BDT	54 (42.9 %)	72 (57.1 %)		
	10000 to 20000 BDT	105 (43.4 %)	137 (56.6 %)		
	20000 to 30000 BDT	0 (0 %)	7 (100 %)		

Note: N = 378, Significant variables are marked with (∗∗).

### 4.3 Influence of risk perception of COVID-19 on the evacuation decision during cyclone

As cyclone Amphan was the fiercest tropical cyclone ever originated in the Bay of Bengal, Bangladesh had done a massive evacuation activity by evacuating around 2 million coastal people in only 14,636 shelters compromising the COVID-19 risk issue (Appadurai, 2020). Though the evacuation percentage during cyclone Amphan is much higher than the previous cyclones, the evacuation scenario of cyclone Amphan was unalike from the previous cyclones due to the risk of COVID-19 pandemic and subsequent nationwide lockdown. The COVID-19 outbreak risk greatly affected the life and livelihood of the coastal people of Bangladesh (Lima et al., 2021). Therefore, unlike the previous cyclone, the coastal people's perception of COVID-19 risk may also influence the evacuation decision during cyclone Amphan. To understand the influence of COVID-19 risk perception on evacuation decisions, a null hypothesis is assumed that COVID-19 risk perception has no influence on coastal people's evacuation decisions during Amphan. Therefore, an independent samples t-test was used to compare the mean of COVIDs-19 risk perception score of evacuee (n = 159) and non-evacuee (n = 219) respondents during cyclone Amphan to analyze the influence of COVID-19 risk perception on evacuation decision (Table 4). Shapiro-Wilk statistics were also employed to measure the normality of COVID-19 risk perception data, and the Shapiro-Wilk statistics were insignificant, indicating that the assumption of normality was not violated. Levene's test was also non-significant; thus, an equal variance can be assumed for both groups, and the null hypothesis is rejected. The t-test was statistically significant, with mean COVID-19 risk perception score of non-evacuee (M = 0.6742, SD = 3.023) was significantly higher (mean difference -1.6, 95 % CI [-2.19, -1.01]), than the evacuee (M = -0.9286, SD = 2.72), t (376) = -5.309, p < .001, two-tailed, Hedges's gs = 0.55. This proves a relationship exists between COVID-19 risk perception and the evacuation behavior of the respondents.

**Table 4:** Mean difference of perception scores of COVID-19 risk between evacuee and non-evacuee during cyclone Amphan.

	Evacuation Status	N	Mean	Standard Deviation	t value
COVID-19 Risk	Evacuee	159	-.9286	2.71772	-5.309∗
Perception Score	Non-Evacuee	219	.6742	3.02087	

∗*p* < 0.05.

The common language (CL) effect size of this study indicates that the chance that for a randomly selected pair of individuals, the COVID-19 risk perception score of a non-evacuee is higher than the score of an evacuee is 65 %. It means if a respondent is randomly chosen from the non-evacuee population, there is a 65 % probability that the respondent has a high-risk perception. So, based on the statistical analysis of the COVID-19 risk perception and evacuation decision data, it can be concluded that the respondents with a high perception score of COVID-19 risk are 65 % less likely to evacuate than the respondent with a low perception score during cyclone Amphan in Bangladesh.

## 5 Discussion

Risk perception, the most complex and dynamic dimension of the social aspect of vulnerability, requires global attention during a pandemic as public concern is the major known weapon to control the pandemic's dispersion (Kuang et al., 2020, He et al., 2020; Mortazavi and Ghardashi, 2021). Since the pandemic declaration, risk perception studies about COVID-19 and its influence or association with every aspect of public health are the major topics of discussion of social scientists (Van Bavel et al., 2020). Despite being one of the important sections of the public health study, no study has been done to understand the influence of COVID-19 in the natural disaster scenario. Decisions taken during the time of disaster are always influenced by the perceived risk of the affected people and policymakers (Samadipour et al., 2020; Mortazavi et al., 2021). Especially when public concern is a major issue at the time of the pandemic, it is extremely important to study the influence of public perception about COVID-19 risk on the affected people's decisions during a disaster scenario. Therefore, this study is one of the first studies to examine the coastal people's perception of COVID-19 risk and its influence on the evacuation decision during cyclone Amphan. During the pandemic, the conventional approach of gathering all people in one safe place during a cyclone is contradictory to the concept of social distancing. From the beginning of 2020, the social distancing (at least 3 ft.) concept is highly promoted by national and international public health organizations to control the pandemic's dispersion (WHO, 2020). Moreover, the cyclone is a common phenomenon for coastal people, but COVID-19 is an unprecedented event. So, when a category 5 cyclone was about to hit the coastal areas of Bangladesh, people had to take the daunting decision of risking life by staying at home or risking infection by evacuating to a crowded safe place. To understand this dilemma of the coastal people of Bangladesh, this study develops a COVID-19 risk perception index using principal component analysis and then analyzes the association of COVID-19 risk perception score with the evacuation decision during cyclone Amphan. This study is conducted based on the primary survey database administered in a coastal district of Bangladesh one month after the landfall of cyclone Amphan. It was identified that people's understanding and perception of risk related to COVID-19 depends on several factors, including cognitive factors, political factors, protective behavior, trust factor, fatality perception factor, religious factors, willingness factors, prejudicial factors, and emotional factors. The respondents were categorized on a very low to very high scale based on the risk perception index score from the data analysis. Though around half of the respondent (45.77 %) falls in the moderate COVID-19 risk perception category, the scale is leaned in the side of higher risk perception rather than the lower side. It is also identified that the respondent's education, age, and income are significantly associated with COVID-19 risk perception. The data analysis also observed that education and income have a proportional relationship with the risk perception score. The most important finding of this research that the respondents with a high perception score of COVID-19 risk are 65 % less likely to evacuate than the respondent with low perception scores during cyclone Amphan in Bangladesh. These findings of risk perception categories and their influence on disaster evacuation provide an important message for public health officials, emergency management experts, and policymakers. The detailed analysis of each factor of COVID-19 risk perception, shown in Figure 4, provides a perspicuous understanding of the gaps and scope of improvements for public health officials. According to Figure 4, some alarming facts are identified that still, 13.8 % of people aren't aware of the COVID-19 dispersion, 6.6 % of people hardly recall the symptoms of COVID-19, 5.8 % of people don't know the process of COVID-19 testing, and 17.7 % people don't think wearing a mask is important. Besides, 19.8 % of people feared that society might boycott them if they get infected by COVID-19, and 28.3 % are anxious about quarantine. Therefore, public health officials should emphasize mass awareness about the risk and change public perception about the aforementioned issues of COVID-19 to control the dispersion. Some shocking religious misbeliefs are also identified, including 7.9 % of people who believe COVID-19 is God's punishment, and 12.4 % believe only religious rituals can prevent COVID-19. These misbeliefs negatively control individual-level protective behavior and thus help the dispersion of the pandemic. The government should take necessary initiatives to help people understand the risk of COVID-19 through electronic media, social media, etc., as media proved to be an essential tool for hazard communication (Ahsan and Khatun, 2020). Involving the religious leaders in disseminating accurate information to mass people can be fruitful to break the misbeliefs regarding COVID-19. As most of the respondents heard rumors about COVID-19, the government should also monitor social media and other sources that spread rumors about the pandemic.

Along with the COVID-19 risk perception details, this research provides important insights into how the pandemic shaped the people's evacuation behavior during a cyclone. This study identified that the respondents with high perception scores of COVID-19 risk are 65 % less likely to evacuate than those with low perception scores during cyclone Amphan in Bangladesh. The finding of this research can provide practical insights into evacuation behavior during the pandemic. Though COVID-19 is an unprecedented event for the world, it is widely accepted that this pandemic will not end soon, and the world needs to adopt this new normal situation for the future. Simultaneously, like cyclone Amphan, natural disasters will continue to hit mankind during this pandemic. Most of our national and international disaster management plans are now obsolete with this new normal scenario. Therefore, it is highly essential to formulate new disaster and emergency management plans considering a pandemic risk.

## 6 Concluding remarks

Bangladesh is having a challenging time as the country has to tackle the community transmission of COVID-19 without any notable health infrastructure, policy, and legislative structures to combat a pandemic. Simultaneously, the country faces tremendous natural disasters, including floods, cyclones, landslides, and others, making the country very susceptible. While the world is struggling for total vaccination of COVID-19, social and behavioral sciences is the main hope for valuable insights to manage the pandemic and its impacts. It is crucial to understand citizens' perceptions about COVID-19 and incorporate the pandemic risk in the emergency disaster management plan to combat unwanted events. This research shed light on the coastal people's risk perception regarding the COVID-19 and revealed how COVID-19 influenced coastal people's evacuation decisions during a cyclone scenario. Though many other reasons can influence coastal people's evacuation decisions, this research statistically identified a significant influence of COVID-19 on coastal people's evacuation decisions. The result shows that 23.3 percent of the coastal people in Bangladesh have a low-risk perception about the COVID-19 pandemic and identified the factor-wise perception, which is a major concern for public health and disaster management officials. Risk communication, community-based awareness program, information dissemination through community people, screening, and controlling rumor-spreading mediums need to be introduced in the rural coastal areas to reduce the knowledge gap about COVID-19. Though the primary objective of cyclone evacuation is to ensure health and safety, the COVID-19 pandemic created a unique challenge for emergency managers, public health officials, and other emergency planners. This research provides an important finding that people with high-risk perception were 65 % less likely to evacuate than those with low perception scores during cyclone Amphan in Bangladesh. Thus, public health officials should emphasize escalating public perception regarding COVID-19 to combat the pandemic, and the findings of this research can provide a comprehensive guideline in this. This research revealed the importance of revising evacuation plans, sheltering options and operations, and proper risk communication during this pandemic. As the study identified that many people refused to go to cyclone shelters to avoid the risk of COVID-19 infection, disaster financing such as forecast-based financing should more be focused on individual and community levels to increase the level of preparedness for both COVID-19 pandemic and disasters. Existing cyclone shelters should be equipped with proper COVID-19 protection equipment and medical supplies. The number of evacuation shelters should be increased in coastal areas to avoiding crowding during the pandemic. Vacant hotel rooms, community centers, government offices, or college dormitories could be used as the evacuation shelter to reduce crowding and maintain proper social distancing. The public health official should increase the number of COVID-19 tests in the coastal disaster-prone areas before the cyclone season or other disaster season, and infected people should be separated in a formal quarantine center to avoid spreading. These measures will provide the coastal people a sense of safety in evacuating the emergency shelter during the disaster. This research is expected to help disaster management officials devise a more effective evacuation plan considering the pandemic to ensure the highest and successful evacuation in any upcoming disaster worldwide.

## Declarations

### Author contribution statement

Md. Shaharier Alam: Conceived and designed the experiments; Performed the experiments; Analyzed and interpreted the data; Contributed reagents, materials, analysis tools or data; Wrote the paper.

Torit Chakraborty: Conceived and designed the experiments; Performed the experiments; Contributed reagents, materials, analysis tools or data.

### Funding statement

This research did not receive any specific grant from funding agencies in the public, commercial, or not-for-profit sectors.

### Data availability statement

Data will be made available on request.

### Declaration of interests statement

The authors declare no conflict of interest.

### Additional information

No additional information is available for this paper.

## Footnotes

1 Taka/BDT = Bangladeshi Currency, 1 Taka = 0.012 United States Dollar.
